# Supplementary material for: Synergy in Cystic Fibrosis Therapies: Targeting SLC26A9
Source: Int J Mol Sci. 2021 Dec 2;22(23):13064. doi: 10.3390/ijms222313064 (PMC8658147; doi:10.3390/ijms222313064)
Supplement: Supplementary file 1 [file ijms-22-13064-s001.zip › Figure Legends.pdf]

Synergy in Cystic Fibrosis therapies: Targeting SLC26A9

Supplementary Figures:

Figure S1 – SLC26A9 expression is delayed over the course of differentiation in CF (F508del/F508del) vs. control pHNE cells. (A) Western Blot showing wt-CFTR and SLC26A9 expression during differentiation (days 0, 7 and 14 in air-liquid interface) in primary human nasal epithelial (pHNE) cells derived from a healthy control. (B) Quantification of CFTR and SLC26A9 protein expression detected by WB in (A). (C) F508del-CFTR and SLC26A9 expression in CF pHNE cells over differentiation and its quantification in (D). Data are normalized to loading control and showed as arbitrary units, mean ± SEM (number of experiments (n) = 3). "*" indicates statistically significant differences (unpaired t-test, $p \leq 0.05$).

Figure S2 – Semi-quantitative PCR to assess SLC26A9 knockdown. (A) Semi-quantitative RT-PCR performed in CFBE F508del-CFTR or (B) 16HBE F508del-CFTR cells stably transfected with either shLuciferase (left) or shSLC26A9 (right). (B, D) Summary of data in (A, C), respectively, where knockdown efficiencies (shown in percentages above bars) were determined by comparing the mRNA levels of SLC26A9 normalized to GAPDH mRNA in the shRNA transfected cells with those in cells transfected with the control shRNA (shLuciferase). Data are normalized to GAPDH and presented as mean ± SEM (n = 3).

Figure S3 – SLC26A9 knockdown lowers both wt- and F508del-CFTR expression in 16HBE cells. (A) Western Blot showing CFTR expression changes due to SLC26A9 knockdown in the presence or absence of different CFTR correctors (VX-809, VX-661, VX-445 or the combination VX-661+VX-445). α-tubulin was used as a loading control. (B) Quantification of F508del-CFTR protein expression obtained in (A), with SLC26A9 knockdown and in the presence of different correctors. From left to right: Band C – fully glycosylated CFTR; Band B – core-glycosylated CFTR; and total protein expression. (C) Quantification of wt-CFTR total protein expression, as well as band B and C, in cells transfected with shSLC26A9. Data are normalized to the loading control and presented as mean ± SEM (n = 3). (*) indicates statistical significance when comparing the same treatment in the presence or absence of shSLC26A9 (unpaired t-test, $p \leq 0.05$).

Figure S4 – SLC26A9 knockdown inhibits endogenous wt-CFTR function and the correction of F508del-CFTR by VX-809 in 16HBE cells. (A) Original Ussing chamber tracings obtained for 16HBE wt-CFTR cells transduced with the control shRNA shLuciferase (left) or shSLC26A9 (right). CFTR was activated by 2 µM Forskolin (Fsk) and 25 µM Genistein (Gen) in the presence of the epithelial sodium channel (ENaC) inhibitor (Amiloride 20 µM) and was inhibited by CFTR-inhibitor 172 (30 µM) added to the luminal side. (B) Original Ussing chamber tracings obtained for cAMP-induced Cl^- currents activated and inhibited as in (A) in the absence (top tracings, black) or in the presence (lower tracings, grey) of 2 µM VX-809 for 16HBE F508del-CFTR cells transfected with shLuciferase (left) or shSLC26A9 (right); (C) Summary of $I_{sc\text{-}eq}$ currents of 16HBE wt-/F508del-CFTR transfected with shLuciferase vs shSLC26A9 and treated with DMSO vs VX-809. Data are represented by mean ± SEM and "*" indicates statistically significant differences (unpaired t-test, $p \leq 0.05$). The number of filters (n) used in the statistical analyses is indicated in the graph. Scale bars in (A) and (B) represent 1 min.

Figure S5 – SLC26A9 overexpression increases both wt- and F508del-CFTR expression in 16HBE cells. (A) Western Blot showing CFTR expression changes due to SLC26A9 overexpression in the presence or absence of different CFTR correctors (VX-809, VX-661, VX-445 or the combination VX-661+VX-445). α-tubulin was used as a loading control. (B) Quantification of F508del-CFTR protein expression obtained in (A), with and without SLC26A9 overexpression (+ Dox, and - Dox, respectively) and in the presence of different correctors. From left to right: Band C, Band B, and total protein expression. (C) Quantification of wt-CFTR expression in cells transfected with SLC26A9 under an inducible promoter. Data are normalized to the loading control and presented as mean ± SEM (n = 3). (*) indicates statistical significance when comparing the same treatment in the presence or absence of Dox (unpaired t-test, $p \leq 0.05$).

Figure S6 – SLC26A9 expression levels affect the polarization of CFBE cells. Transepithelial resistance (TEER) measurements of CFBE cells with different levels of SLC26A9 expression over 5 to 7 days in liquid-liquid interface (LLI). TEER of CFBE wt-CFTR (A) or CFBE F508del-CFTR (B) stably expressing shLuc (control) vs shSLC26A9. (C) TEER of SLC26A9 overexpressing CFBE F508del-CFTR cells in the presence or absence of Dox. (*) indicates significant difference (unpaired t-test, $p \leq 0.05$). The number of filters used in the statistical analyses is indicated in each graph.
