# Supplementary material for: Synergy in Cystic Fibrosis Therapies: Targeting SLC26A9
Source: Int J Mol Sci. 2021 Dec 2;22(23):13064. doi: 10.3390/ijms222313064 (PMC8658147; doi:10.3390/ijms222313064)

Figure S1

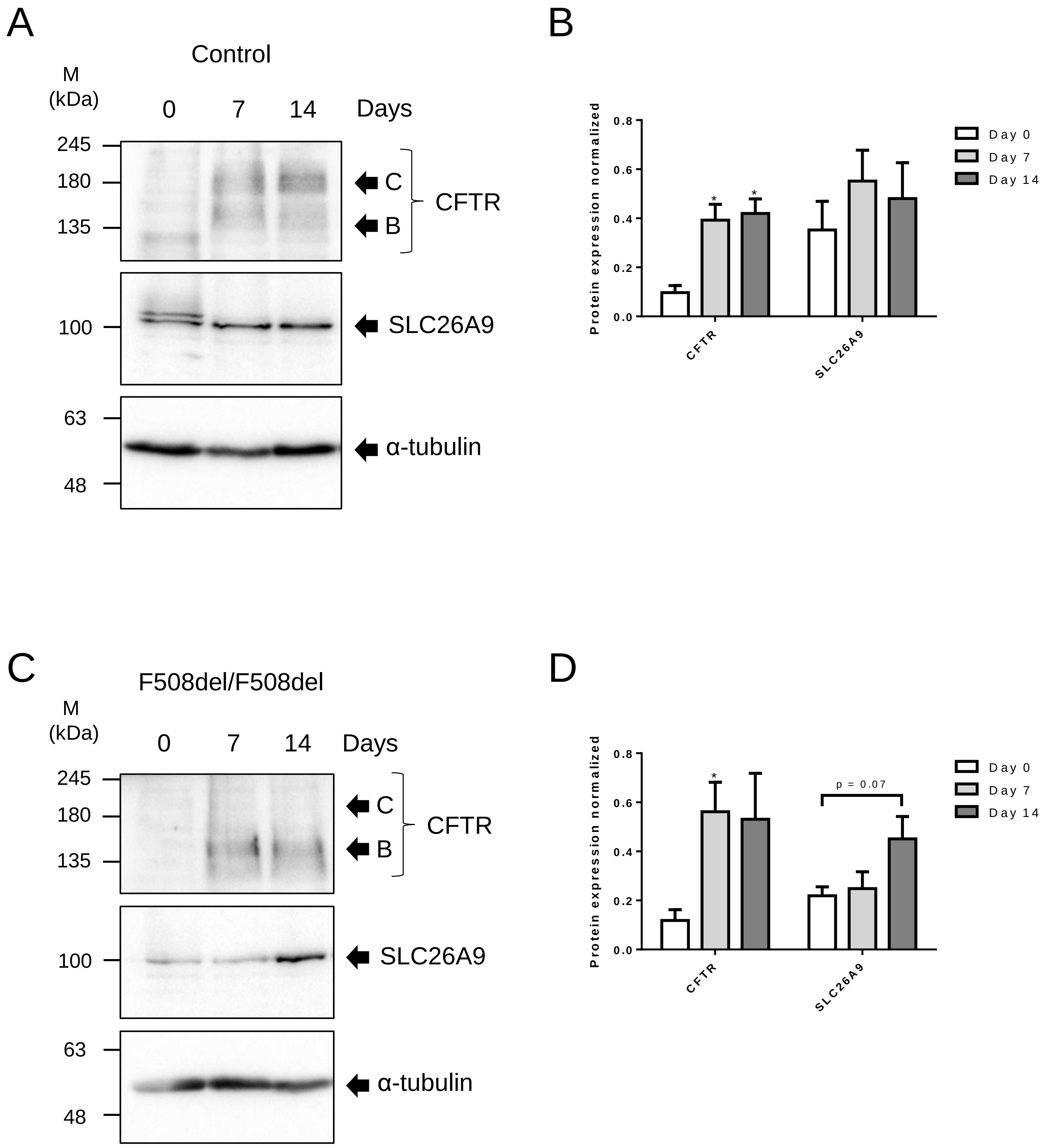
A
Control
M
(kDa)
0
7
14
Days
245
180
135
100
63
48
C
B
CFTR
SLC26A9
α-tubulin
B
Protein expression normalized
0.0
0.2
0.4
0.6
0.8
*
*
CFTR
SLC26A9
Day 0
Day 7
Day 14
C
F508del/F508del
M
(kDa)
0
7
14
Days
245
180
135
100
63
48
C
B
CFTR
SLC26A9
α-tubulin
D
Protein expression normalized
0.0
0.2
0.4
0.6
0.8
*
p = 0.07
CFTR
SLC26A9
Day 0
Day 7
Day 14

Figure S2

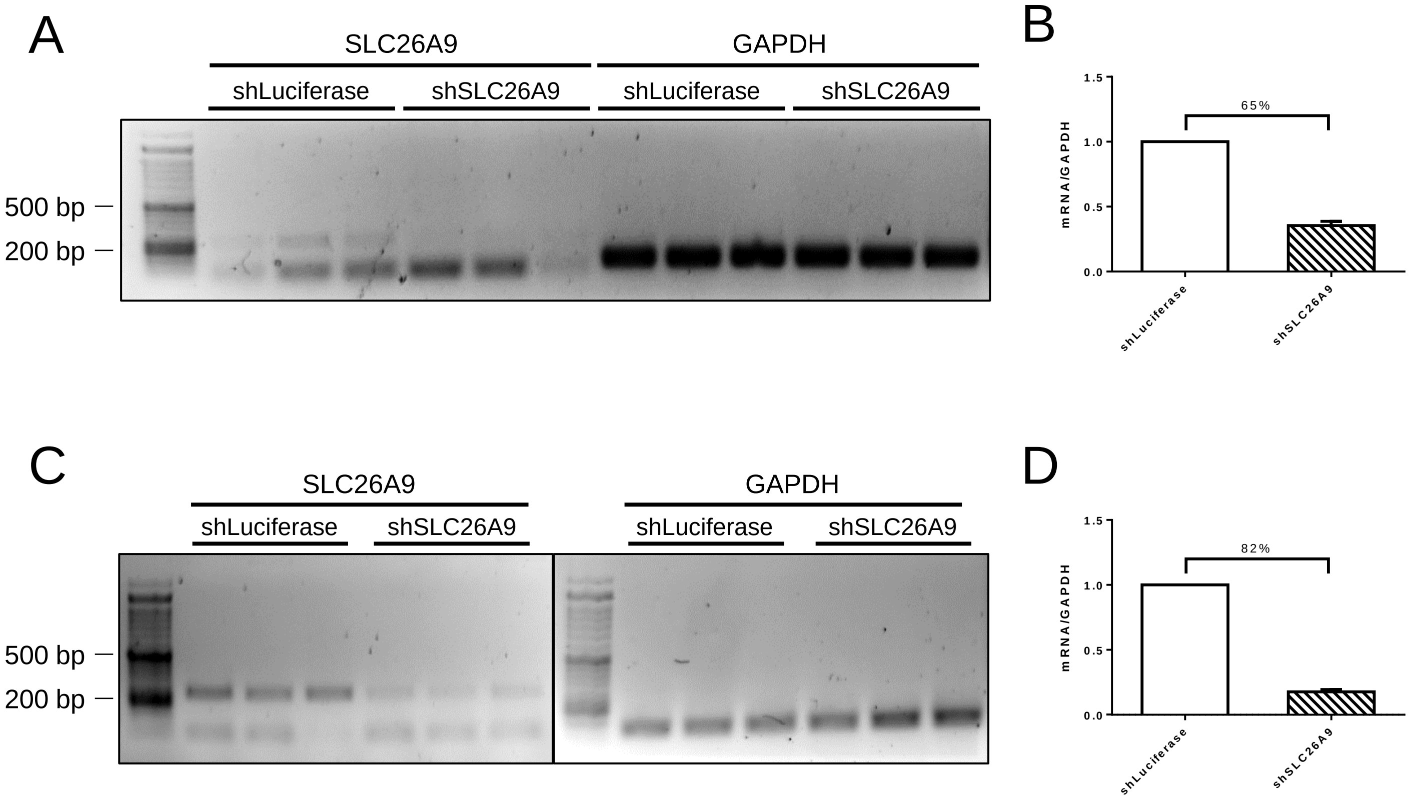

A
SLC26A9
GAPDH
shLuciferase
shSLC26A9
shLuciferase
shSLC26A9
500 bp
200 bp
B
1.5
1.0
0.5
0.0
mRNA/GAPDH
65%
shLuciferase
shSLC26A9
C
SLC26A9
GAPDH
shLuciferase
shSLC26A9
shLuciferase
shSLC26A9
500 bp
200 bp
D
1.5
1.0
0.5
0.0
mRNA/GAPDH
82%
shLuciferase
shSLC26A9

Figure S3

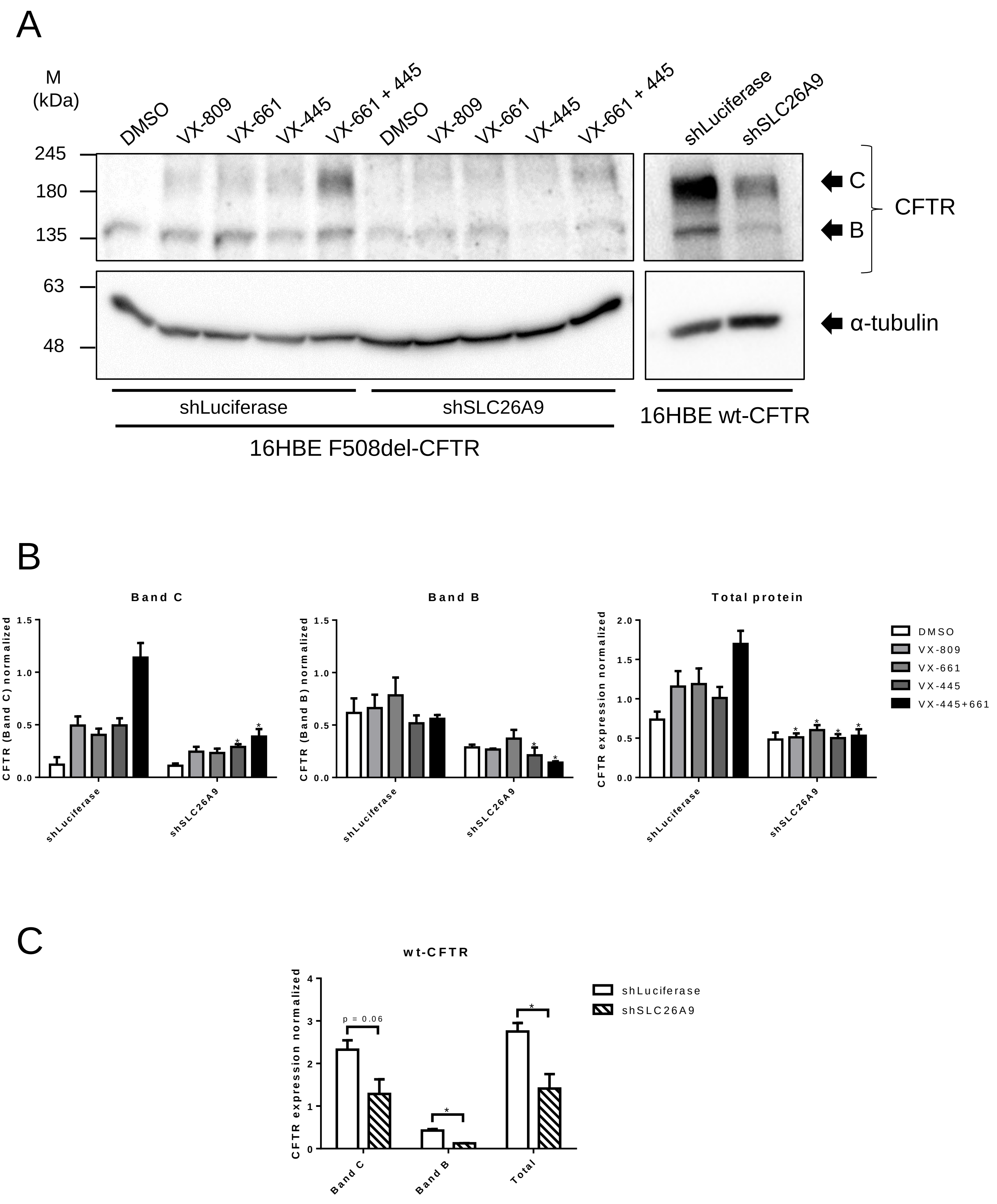
A
M (kDa)
DMSO
VX-809
VX-661
VX-445
VX-661 + 445
DMSO
VX-809
VX-661
VX-445
VX-661 + 445
shLuciferase
shSLC26A9
245
180
135
63
48
C
B
CFTR
α-tubulin
shLuciferase
shSLC26A9
16HBE F508del-CFTR
16HBE wt-CFTR
B
Band C
CFTR (Band C) normalized
Band B
CFTR (Band B) normalized
Total protein
CFTR expression normalized
shLuciferase
shSLC26A9
DMSO
VX-809
VX-661
VX-445
VX-445+661
C
wt-CFTR
CFTR expression normalized
p = 0.06
Band C
Band B
Total
shLuciferase
shSLC26A9

Figure S4

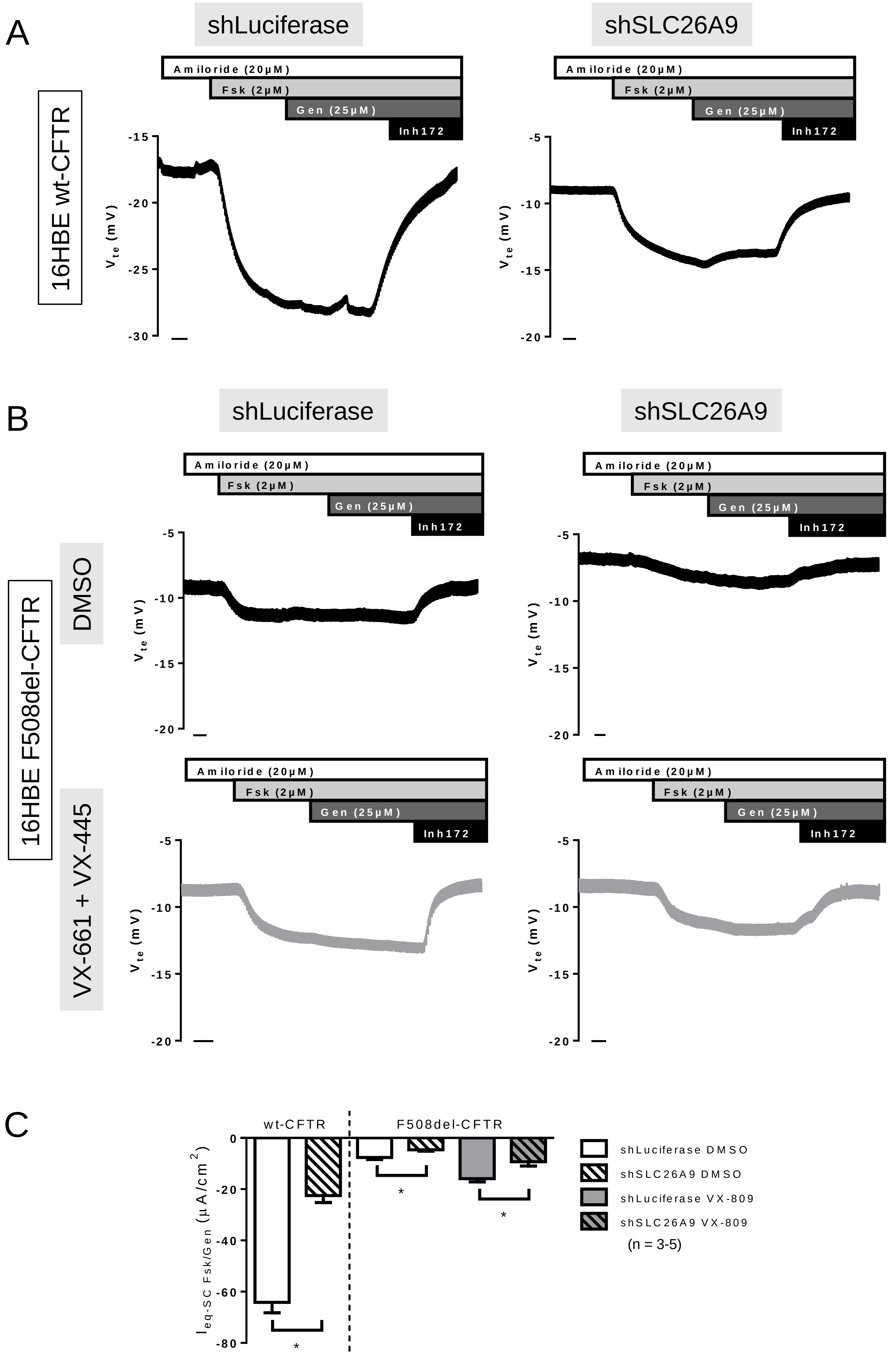
A
shLuciferase
shSLC26A9
16HBE wt-CFTR
Amiloride (20μM)
Fsk (2μM)
Gen (25μM)
Inh172
Vte (mV)
-15
-20
-25
-30
-5
-10
-15
-20
B
shLuciferase
shSLC26A9
16HBE F508del-CFTR
DMSO
VX-661 + VX-445
Amiloride (20μM)
Fsk (2μM)
Gen (25μM)
Inh172
Vte (mV)
-5
-10
-15
-20
C
wt-CFTR
F508del-CFTR
Ieq-SC Fsk/Gen (μA/cm2)
0
-20
-40
-60
-80
*
shLuciferase DMSO
shSLC26A9 DMSO
shLuciferase VX-809
shSLC26A9 VX-809
(n = 3-5)

Figure S5

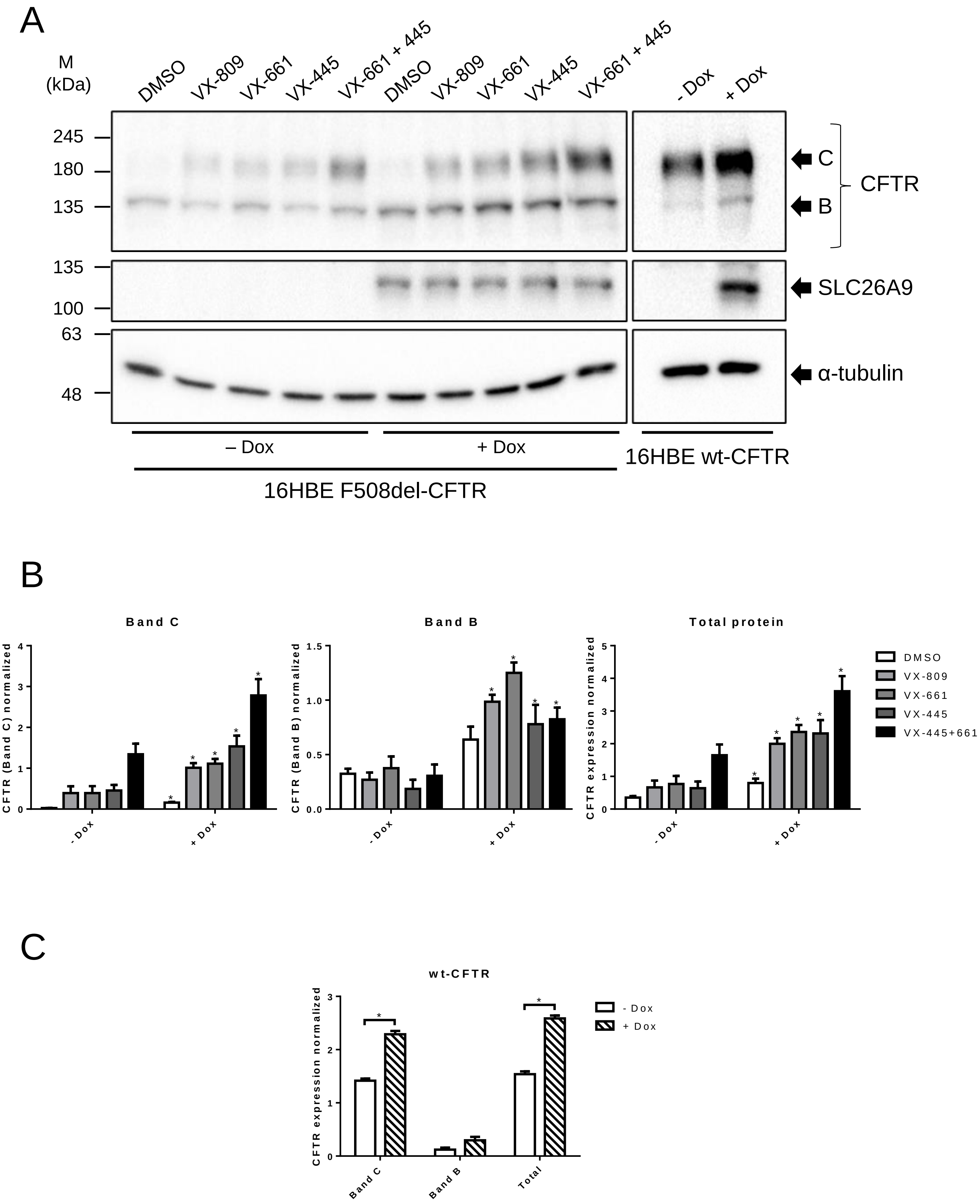
A
M (kDa)
DMSO
VX-809
VX-661
VX-445
VX-661 + 445
DMSO
VX-809
VX-661
VX-445
VX-661 + 445
- Dox
+ Dox
245
180
135
135
100
63
48
C
B
CFTR
SLC26A9
α-tubulin
– Dox
+ Dox
16HBE wt-CFTR
16HBE F508del-CFTR
B
Band C
CFTR (Band C) normalized
Band B
CFTR (Band B) normalized
Total protein
CFTR expression normalized
- Dox
+ Dox
DMSO
VX-809
VX-661
VX-445
VX-445+661
C
wt-CFTR
CFTR expression normalized
Band C
Band B
Total
- Dox
+ Dox

Figure S6

A

SLC26A9 knockdown

CFBE wt-CFTR

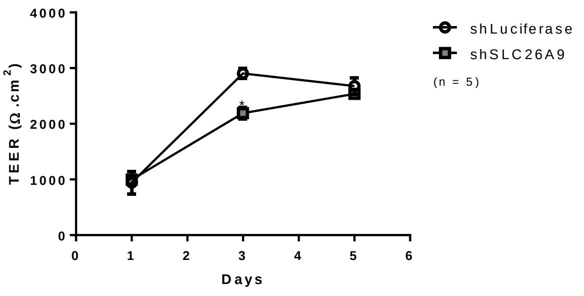

B

CFBE F508del-CFTR

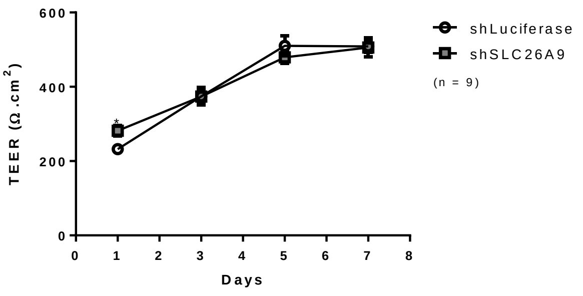

C

SLC26A9 overexpression

CFBE F508del-CFTR

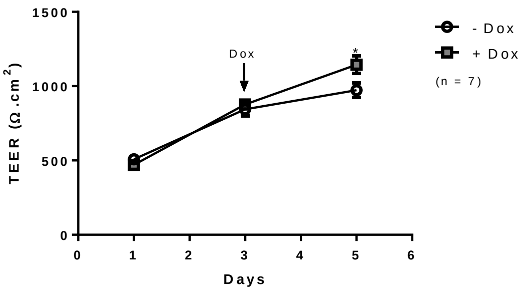

Supplement: Supplementary file 1 [file ijms-22-13064-s001.zip › Sup.Figures_Pinto et al.pdf]
